# Synthesis of Nitrogen and Phosphorus/Sulfur Co-Doped Carbon Xerogels for the Efficient Electrocatalytic Reduction of p-Nitrophenol

**DOI:** 10.3390/ijms24032432

**Published:** 2023-01-26

**Authors:** Chaolong Wang, Dengxia Zhu, Huiting Bi, Zheng Zhang, Junjiang Zhu

**Affiliations:** Hubei Key Laboratory of Biomass Fibers and Eco-Dyeing & Finishing, College of Chemistry and Chemical Engineering, Wuhan Textile University, Wuhan 430200, China

**Keywords:** p-nitrophenol, carbon xerogels, synergistic co-doping, electrocatalytic reduction

## Abstract

Carbon xerogels co-doped with nitrogen (N) and phosphorus (P) or sulfur (S) were synthesized and employed as catalysts for the electrocatalytic reduction of p-nitrophenol (p-NP). The materials were prepared by first synthesizing N-doped carbon xerogels (NDCX) via the pyrolysis of organic gels, and then introducing P or S atoms to the NDCX by a vapor deposition method. The materials were characterized by various measurements including X-ray diffraction, N_2_ physisorption, Transmission electron microscopy, Fourier Infrared spectrometer, and X-ray photoelectron spectra, which showed that N atoms were successfully doped to the carbon xerogels, and the co-doping of P or S atoms affected the existing status of N atoms. Cyclic voltammetry (CV) scanning manifested that the N and P co-doped materials, i.e., P-NDCX-1.0, was the most suitable catalyst for the reaction, showing an overpotential of −0.569 V (vs. Ag/AgCl) and a peak slop of 695.90 μA/V. The material was also stable in the reaction and only a 14 mV shift in the reduction peak overpotential was observed after running for 100 cycles.

## 1. Introduction

Nitrophenol (p-NP) is an important industrial raw material and has been widely used in the synthesis of pesticides, medicines, synthetic dyes, and so on [1,2,3]. Meanwhile, it is also a severe environmental pollutant discharged in wastewater, posing a threat to rivers, soil, and groundwater [4,5,6], and to the human nervous and blood systems by hindering the formation of methemoglobinemia [6,7]. Indeed, p-NP has been listed as one of the 129 priory control pollutants by the USA Environmental Protection Agency due to its severe threats to human health. The severe toxicity, high stability, and large solubility in water make the removal of p-NP in wastewater a challenging topic in the field of environmental protection.

Physical absorption, biodegradation, and chemical degradation are three primary approaches for the treatment of p-NP in wastewater [8,9,10,11], and the chemical approaches, e.g., Fenton oxidation [12,13], ozone oxidation [14], photochemical degradation [15], and chemical reduction [16], are of special interest, as they can completely degrade the p-NP into CO_2_ and H_2_O compared to the physical adsorption and they are more facilitating and cheap compared to biodegradation. However, the chemical operation requires the use of powerful and costly oxidants, and the process produces large amounts of residual contaminants [16,17,18,19], causing secondary pollution. For these reasons, electrochemical reduction has been considered as an alternative technology for the removal of p-NP owing to its high sensitivity, simple equipment, convenient operation, and environmentally friendly process [17]. Moreover, the toxicity of the reduction product, p-hydroxyaminophenol, is 1/500 less than that of p-NP [20,21,22].

Metal-free carbon materials, e.g., carbon fiber, carbon nanotube, carbon xerogels, and graphene have received great attention in electrocatalysis owning to their low price, high stability, large surface area, various pore architectures, abundant functional groups, and good recyclability [23,24,25,26,27]. Among them, carbon xerogel has attracted special interest due to its outstanding performance, including: (1) the large surface area contributing to more active sites for the electrocatalytic reduction of p-NP; (2) the controllable structure and tunable surface properties which can adjust the ion transport rate in accordance with the requirements; (3) compared with other carbon materials, carbon xerogel has remarkable electrochemical stability and conductivity [28,29,30]. It has also been suggested that the electrical conductivity, wettability, and interface compatibility of carbon-based materials could be improved by doping heteroatoms, benefiting the contact between the electrolyte and the electrode [31,32,33,34,35,36]. Moreover, because of the covalent structure, carbon can accommodate numerous non-metallic atoms such as N, P, S, O, etc., in the matrix through a simple carbonization or vapor deposition process, adjusting the electronic and geometric properties and exposing more active sites on the surface. For example, synergistic effects can be produced by uniting two or more heteroatoms (N/P, N/S, or N/P/S) with different electronegativities, contributing to the electrocatalytic activity [37,38,39,40,41,42].

Herein, we report the synthesis of intelligent and heteroatomic-doped 2D carbon xerogels, with N as the primary dopant, and P or S as the secondary elements. It can increase the number of active sites of N-doped carbons xerogels and accomplish the “synergistic coupling”, enhancing the electrocatalytic activities of carbons xerogels. The evaluation of the systematic promotional effects induced by N,P- and N,S- co-doped carbon xerogels were investigated by a series of characterizations and electrocatalytic experiments. Firstly, the amounts of N atoms doped in carbon xerogels were optimized via a measurement of the electrocatalytic p-nitrophenol reduction reaction. Secondly, the applicable P or S doping based on the above N-doped carbon xerogels was explored. The results revealed the optimal doping of N, P, and S and the most appropriate couple (N,P- or N,S-) of carbon xerogels was elected for the electrocatalytic reduction of p-NP.

## 2. Results and Discussion

### 2.1. Characterization of the Samples

**Scheme 1:**
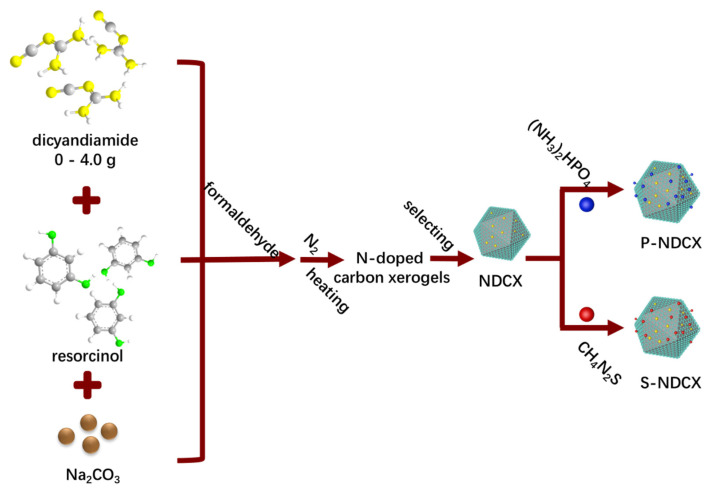
Fabrication of the N-, N,P-, and N,S-doped carbon xerogels.

The synthetic process of N,P- or N,S- co-doped carbon xerogels is shown in Scheme 1. In the procedure, the resorcinol and formaldehyde undergo polymerization and cross-linking reactions, and the Na_2_CO_3_ promotes the reaction of resorcinol and formaldehyde by accelerating the deprotonation of resorcinol [43]. The mass of dicyandiamide added in the synthesis process determines the quantity of N doped in the carbon xerogels. The CVD process introduces P/S atoms to the surface of NDCX. Meanwhile, the NH_3_, NO_2_, SO_2_, H_2_S, CO_2_, and other gases released from the precursors during the heating treatment, generate micropores and improve the porosity and surface area of the doped carbon xerogels [44].

**Figure 1:**
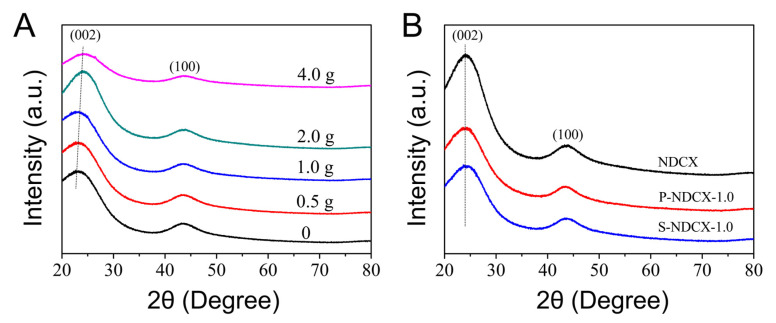
XRD patterns of (**A**) N-doped carbon xerogels prepared with 0–4.0 g of dicyandiamide and (**B**) NDCX, P-NDCX-1.0, and S-NDCX-1.0.

XRD patterns showed that all samples exhibited two large and broad diffraction peaks at 2θ ≈ 23° and 44°, as shown in Figure 1, which are attributed to the (002) and (100) lattice plane of amorphous carbon with low crystallinity [44,45]. For N-doped carbon xerogels, as shown in Figure 1A, the peak position of the (002) plane was red-shifted with the increase in the amount of dicyandiamide, accompanied by a decrease in peak intensity, which can be ascribed to the narrow C-C(N) distance caused by the doping of N atoms with a smaller atomic radius [46]. This proves that N atoms were successfully doped in the carbon skeleton. For P-NDCX-1.0 and S-NDCX-1.0, as shown in Figure 1B, the peak position remained after the co-doping of P or S, indicating that no change in the matrix of NDCX occurred, since their doping occurred mainly on the surface of NDCX.

**Figure 2:**
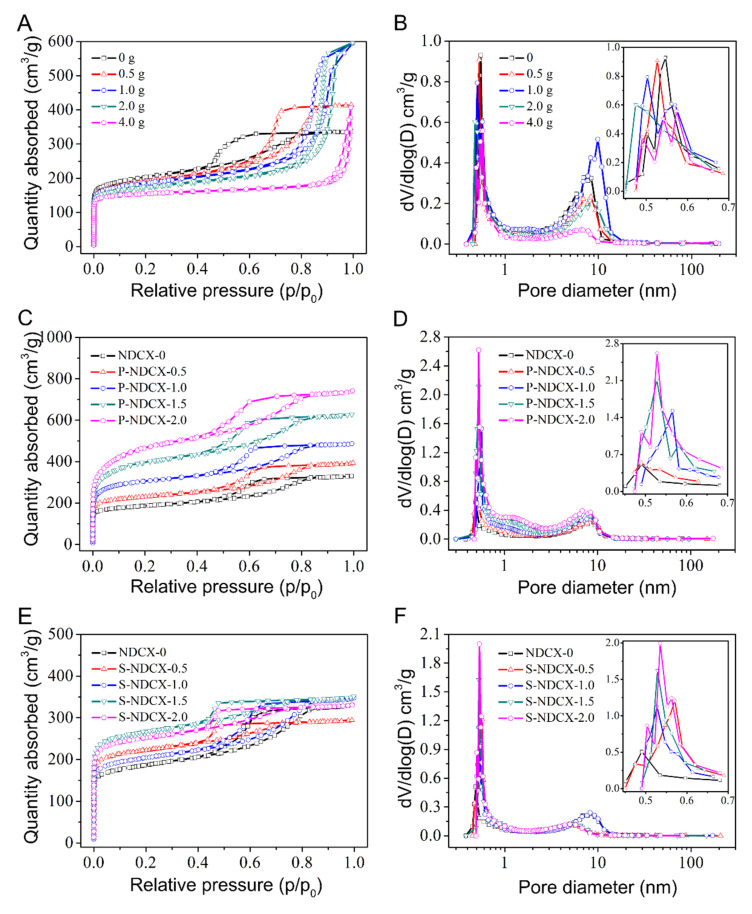
N_2_ sorption isotherms (**A**,**C**,**E**) and pore distributions (**B**,**D**,**F**) of carbon xerogels with different amounts of dicyandiamide, P-NDCX-m, and S-NDCX-m.

To evaluate the surface area and pore size distribution of the N, N,P-, and N,S-doped carbon xerogels, N_2_ physisorption measurements were performed. For carbon xerogels doped with different amounts of N atoms, the abrupt increase in the N_2_ adsorption quantity at *p*/*p*_0_ < 0.05 and the obvious hysteresis loop observed in the curves indicate that both micropores and mesopores were present in the materials, as shown in Figure 2A. However, it was noted that the dicyandiamide amount significantly affected the shape of isotherms, in that the hysteresis loop moved to a high *p*/*p*_0_ with the increase in dicyandiamide, which suggests that more of the large mesopores were produced, similar to what has been observed elsewhere [47]. The movement of the hysteresis loop and the change of isothermal shape verifies that different amounts of N atoms were doped to the carbon matrix and changed the textural structure. Note that for sample prepared with excess amounts of dicyandiamide (e.g., 4.0 g), the pore volume and pore size significantly decreased, as shown in Figure 2B, which may have been due to the excess amount of N atoms doped into the carbon framework, altering the original matrix structure. Considering the large changes in the textural structure, and together with the catalytic activity tested in the following experiments, we selected the carbon xerogels doped with 2.0 g dicyandiamide, i.e., NDCX, for further investigation.

After the co-doping of P and S atoms on NDCX, the position and shape of the hysteresis were restored to that of the materials with low N doping, as shown in Figure 2C,E. This may be attributed to high temperature NDCX reprocessing, which removes excess N atoms from the matrix. To support this assumption, we re-heated the NDCX in N_2_ atmosphere without presenting the (NH_3_)_2_HPO_4_ or CH_4_N_2_S precursor, and found that the re-heated sample, defined as NDCX-0, indeed showed a restored position and shape for the hysteresis loops. Hence, it is suggested that the doping of N atoms on the carbon framework should be optimized to maintain the matrix structure. Because of the release of excess N atoms, both the pore volume and pore size of the P-NDCX-1.0 or S-NDCX-1.0 increased, compared with those of the NDCX, as shown in Figure 2D,F and Table 1.

While the process of doping either P or S atoms restored the matrix structure of NDCX, changes in the surface area, pore volume, and pore diameter were different. For example, we selected P-NDCX-1.0 and S-NDCX-1.0 with good catalytic performance for p-NP electroreduction (see below) for comparison. P-NDCX-1.0 showed a larger surface area (989.3 m^2^/g), pore volume (0.75 m^3^/g), and mesopore size (8.31 nm) than S-NDCX-1.0, indicating that the doping of P atoms had a greater effect than that of the S atoms. In fact, doping a larger number of P atoms may enhance the textural properties of NDCX. For example, the P-NDCX-2.0, prepared by addition of 2.0 g (NH_3_)_2_HPO_4_ to NDCX, exhibited a surface area of up to 1522.1 m^2^/g and a pore volume of 1.14 m^3^/g, as shown in Table 1.

**Figure 3:**
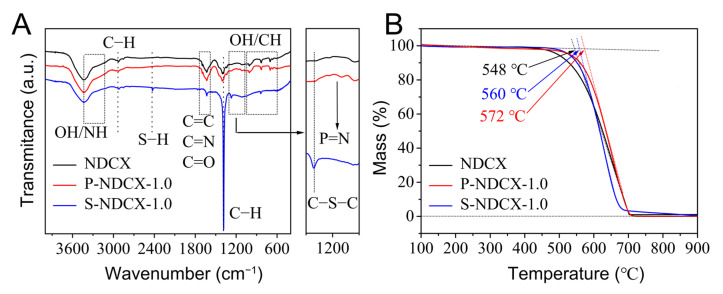
(**A**) FT-IR spectra and (**B**) TGA profiles of the NDCX, P-NDCX-1.0 and S-NDCX-1.0.

FT-IR spectra of the three selected samples, NDCX, P-NDCX-1.0, and S-NDCX-1.0, are illustrated in Figure 3A. According to the literature [48,49,50], the peaks at 3145–3429 cm^−1^ are assigned to the -OH and N-H absorption band; the peaks at 2921 cm^−1^ and 1400 cm^−1^ are assigned to the stretching vibration of C-H bond; the peaks at 1635–1700 cm^−1^ are assigned to the vibration of the double bond of C=C/C=N/C=O groups; and the peaks at 1000 cm^−1^ are assigned to the vibration of -OH and C-H bond. These results suggest that N atoms were spiked to the carbon framework, accompanied with the formation of some surface groups. In addition, because of the co-doping of P or S atoms, an additional peak at 1150 cm^−1^, attributed to the stretching vibration of P=N bond, was observed for P-NDCX-1.0, and two extra peaks at 1390 cm^−1^ and 2422 cm^−1^, attributed to the vibration of C-S-C and S-H bond, were observed for S-NDCX-1.0. This shows that P or S were successfully integrated into the NDCX framework.

The TGA profiles (Figure 3B) show that the materials started to burn at temperatures above 450 °C and were finished at 700 °C. The burning temperature of NDCX was slightly lower than that of P-NDCX-1.0 and S-NDCX-1.0, indicating that the doping of P or S improved the stability of NDCX, which could be due to the change in textural structure, as discussed above. For all the samples, no residue was detected after 700 °C, suggesting that they were completely oxidized and released as gases.

**Figure 4:**
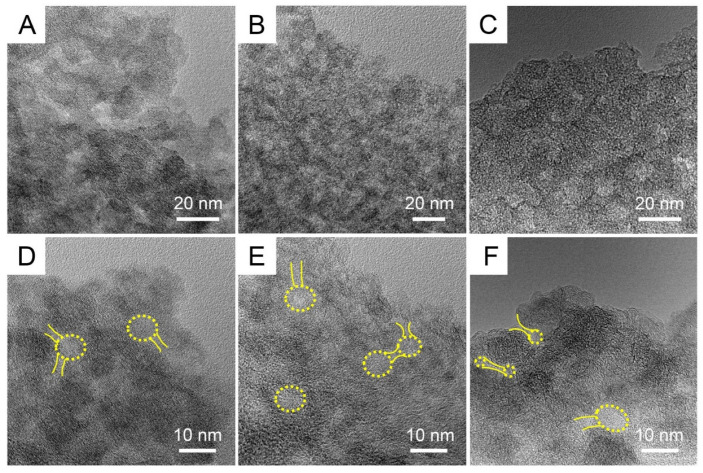
TEM images of (**A**,**D**) NDCX, (**B**,**E**) P-NDCX-1.0, and (**C**,**F**) S-NDCX-1.0 with different magnification.

The different magnification TEM images show that the materials had microporous structures (the micropores are marked in yellow), which were more pronounced for P-NDCX-1.0 and S-NDCX-1.0, as shown in Figure 4. The presence of micropores guaranteed the increase in surface area, which is a crucial factor affecting the efficient contact between the catalyst and the electrolyte, and hence the electron transporting rate between them.

**Figure 5:**
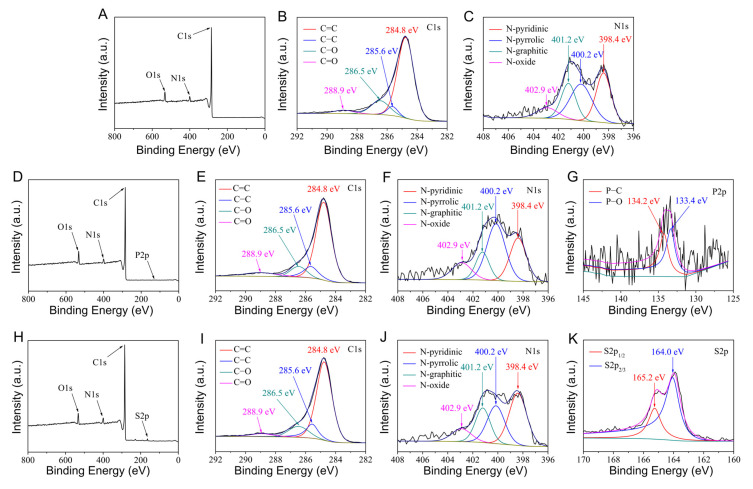
(**A**) the whole XPS surveys, (**B**) C1s, and (**C**) N1s spectrum of NDCX; (**D**) the whole XPS surveys, (**E**) C1s, (**F**) N1s, and (**G**) P2p spectrum of P-NDCX-1.0; (**H**) the whole XPS surveys, (**I**) C1s, (**J**) N1s, and (**K**) S2p spectrum of S-NDCX-1.0.

XPS measurements were conducted to detect the surface element composition and valence state of the materials, as shown in Figure 5. For NDCX, the whole spectrum showed the presence of C, N, and O atoms on the surface (Figure 5A), which was in line with the results of FTIR spectra. The C1s spectrum could be fitted to four peaks that could be attributed to carbon in C=C (284.8 eV), C−C (285.6 eV), C−O (286.5 eV) and C=O (288.9 eV) [37,39,51], respectively, as shown in Figure 5B,E,I. It is worth noting that the main peak of C negligibly shifted among them, as the amount of P or S doped in the material was lower and hardly affected the binding energy of carbon. The N1s spectrum could also be deconvoluted into four peaks, with the binding energy at 398.4 eV, 400.2 eV, 398.4 eV, and 402.9 eV attributed to N-pyridinic, N-pyrrolic, N-graphitic, and pyridine N-oxide [47,52,53], respectively, as shown in Figure 5C.

For P-NDCX-1.0, the whole XPS spectrum showed the signal of P atom as desired, in addition to that of the C, N, and O atoms, as shown in Figure 5D. By comparison, it was found that the co-doping of P and N increased the proportion of N-oxide and N-pyrrolic, which are general defect sites in the carbon skeleton and beneficial to electron transport [44], as shown in Figure 5F. Deconvolution on the P2p spectrum showed two peaks at a binding energy of 134.2 eV and 133.4 eV, which can be assigned to P−C and P−O [37], respectively, as shown in Figure 5G. In addition, the peak area of P−C was almost equal to that of the P−O, which indicated that a portion of P atoms were incorporated into the carbon framework [54].

Similarly, the whole spectrum of S-NDCX-1.0 showed the existence of C, N, O, and S atoms, Figure 5G. The co-doping of S and N significantly increased the percentage of surface N atoms, as shown in Figure 5H and Table 2. The two fitted peaks of S2p at a binding energy of 164.0 eV and 165.2 eV were assigned to S2p_3/2_ and S2p_1/2_, respectively, as shown in Figure 5I, which implied the existence of the C-S bond [55] in the material. The results confirmed the successful doping of NDCX.

### 2.2. Electrocatalytic Activity

**Figure 6:**
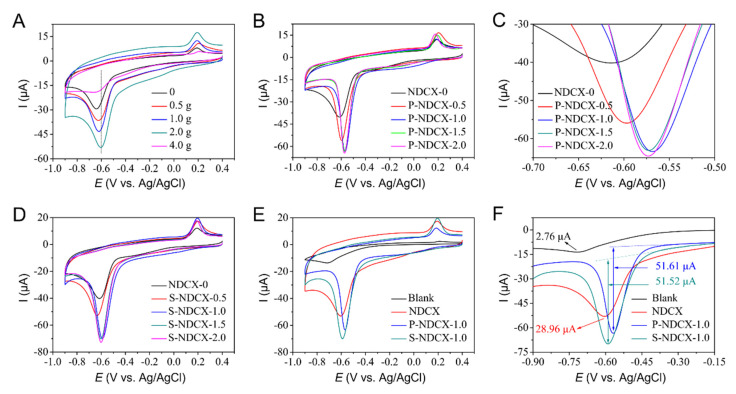
CV curves of (**A**) N-doped carbon xerogels prepared with 0–4.0 g of dicyandiamide, (**B**,**C**) P-NDCX-m prepared with 0.5–4.0 g of (NH_3_)_2_HPO_4_, (**D**) S-NDCX-m prepared with 0.5–2.0 g of CH_4_N_2_S, and (**E**,**F**) Blank GCE, NDCX, P-NDCX-1.0 and S-NDCX-1.0.

Figure 6A presents the CV curves of N-doped carbon xerogels, prepared with different amounts of dicyandiamide, for the electrocatalytic reduction of p-NP. The results show that the material prepared with the addition of 2.0 g dicyandiamide, defined as NDCX, exhibited the lowest overpotential (−0.605 V vs. Ag/AgCl) and the largest reduction current compared with other samples (see line “d” in the picture). This indicates that the doping of appropriate nitrogen can enhance the catalytic ability of carbon xerogel by altering its electronic properties (nitrogen has one more electron than carbon) and textural structures (e.g., formation of more mesopores), as discussed above. When few dicyandiamides were added, the effect of N doping on the electronic properties and textural structures of carbon xerogels was slight, leading to a lower contribution to catalytic activity, while the addition of excess dicyandiamide would result in dicyandiamide polycondensation, pore collapse, and a reduction in the surface area of carbon xerogels, and finally lower the catalytic activity. As a result, the NDCX was selected for a more in-depth investigation in the following.

It has been reported that the doping of P or S is efficient to improve the catalytic performances of carbon materials [56,57,58]. For this reason, the doping of P or S onto the NDCX was conducted. Figure 6B,C shows that the doping of P improved not only the overpotential but also the current intensity, suggesting that the P doping generated more active sites and they had a stronger reactivity. The optimal catalyst was obtained at P-NDCX-1.0, which exhibited an overpotential of −0.569 V (vs. Ag/AgCl) and a reduction current of 51.61 µA. In addition, P-NDCX-1.5 and P-NDCX-2.0 were similar to that of P-NDCX-1.0, indicating that the addition of a 1.0 g (NH_3_)_2_HPO_4_ precursor was sufficient to enhance the catalytic performances of NDCX in the current study. Although the doping of S atoms also strengthened the current of NDCX for p-NP reduction, the decrease in overpotential was less, as shown in Figure 6D. 

The contrasting experiments (Figure 6E,F) show that the blank GCE only exhibited a small reduction peak with a high overpotential (−0.7 V vs. Ag/AgCl), indicating that the direct reduction of p-NP is hard if no catalyst is present. Hence, the reduction peak observed in the presence of catalyst was caused by a catalytic process. The CV curve of P-NDCX-1.0 showed the largest reduction peak and the lowest overpotential, which indicates a larger reduction peak slop and hence a faster reaction rate. From Table 3, which summarizes the electrocatalytic parameters obtained from the CV curves, it can be inferred that the electrocatalytic ability of the materials for p-NP reduction followed the order of P-NDCX-1.0 > S-NDCX-1.0 > NDCX.

The superiority of P-NDCX-1.0 for the electrocatalytic reduction of p-NP was further illuminated by a comparison with other materials reported in the literature, as listed in Table 4. The results show the P-NDCX-1.0 exhibited a small overpotential for the reaction compared with other carbon-based catalysts reported in the literature, showing that P-NDCX-1.0 is a potential catalyst for the electrocatalytic reduction of p-NP.

**Figure 7:**
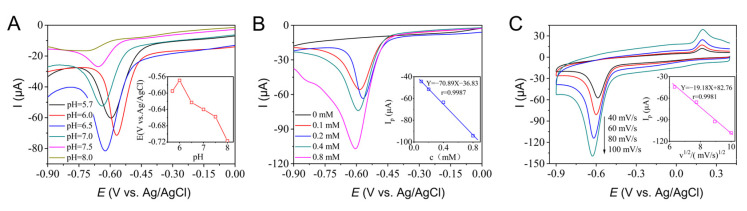
Reduction peak curves at different pH (**A**), p-NP concentration (**B**), and scanning rate (**C**) of P-NDCX-1.0/GCE.

To investigate the role of protons in the electrocatalytic reduction of p-NP, the reduction peaks of P-NDCX-1.0 in PBS were measured with 0.2 mM p-NP at different pH values (5.7~8.0), as shown in Figure 7A. The results show that at pH = 6.0, the P-NDCX-1.0 exhibited the lowest overpotential in the CV curve, suggesting that the pH of 6.0 is the best environment for p-NP reduction. With the further increase in pH, the number of protons decreased and the overpotential shifted to negative position, which indicates that protons were involved in the process of electrocatalytic reduction of p-NP. Inversely, at a lower pH (5.7), the overpotential rose due to the agglomeration of catalysts [4] and hence led to insufficient protons on the surface of the catalyst utilized for the reduction of p-NP.

The reduction peaks of P-NDCX-1.0 in PBS with different p-NP concentrations (0~0.8 mM) were measured at pH = 6.0. Figure 7B shows that the reduction peak current linearly decreased with the p-NP concentration, suggesting that the reduction of p-NP under this condition is a mass transfer-controlled process. In addition, it was found that the reduction peak of P-NDCX-1.0 shifted to a negative position with the increase in scanning rate (40, 60, 80, 100 mV/s), and the peak current was linear to the square root of scanning rate, as shown in Figure 7C. This again confirms that the electrocatalytic p-NP reduction is a mass transfer-controlled process.

**Figure 8:**
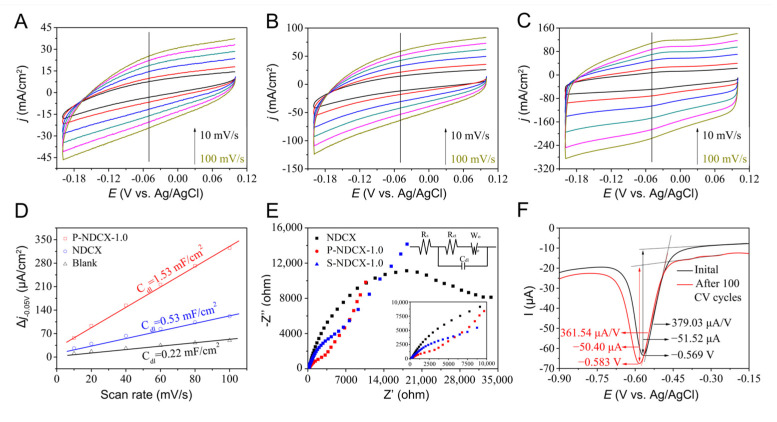
CV curves of (**A**) blank GCE, (**B**) NDCX, and (**C**) P-NDCX-1.0 at different scanning rates in the non-Faradic interval; (**D**) *C_dl_* of P-NDCX-1.0 (*R*^2^ = 0.9992), NDCX (*R*^2^ = 0.9982), and blank GCE (*R*^2^ = 0.9975); (**E**) Nyquist plots of NDCX, P-NDCX-1.0, and S-NDCX-1.0; (**F**) The reduction peak curves of P-NDCX-1.0 before and after 100 CV cycles.

Electrochemically active surface area (ECSA) represents the active site or area that accounts for the Faradaic current transfer during the electrocatalytic process [62]. The ECSA can be calculated from the electric double layer capacitance (*C_dl_*). First, the CV scanning was conducted at different scanning rates (10, 20, 40, 60, 80, and 100 mV/s) in the potential range of 0.1~−0.2 V (vs. Ag/AgCl, without any Faradaic current). After that, the charge–discharge current density difference at −0.05 V vs. Ag/AgCl (Δ*j*_−0_._05V_ = *j_a_* − *j_c_*) was plotted as a function of the scanning rate, and the slope was calculated by linear fitting according to the equation: “*i_c_ = v·C_dl_*”. Finally, the *C_dl_* was converted to ECSA according to the equation: ECSA = *C_dl_*/*Cs* [62,63,64]. The *C_dl_* of blank GCE, NDCX, and P-NDCX-1.0 thus obtained was 0.22, 0.53, and 1.53 mF/cm^2^, respectively, as shown in Figure 8A–D, which shows that the ECSA of P-NDCX-1.0 was about 7 times larger than that of the blank GCE. This could be a reason why the P-NDCX-1.0 sample exhibited a high performance for the reaction.

Electrochemical impedance spectrum (EIS) can reflect the contact and charge-transfer impedance during the reduction process of p-NP, conforming to a better electrochemical activity with a smaller impedance. The electrochemical processes of NDCX, P-NDCX-1.0, and S-NDCX-1.0 contained the kinetic diffusion process in the high frequency region and the Warburg diffusion control process in the low frequency region. After doping with P, P-NDCX-1.0 had a smaller radius in the high frequency region and a shorter line in the low frequency region, as shown in Figure 8E. This indicates that the charge transfer resistance (Rct) and Warburg impedance were smaller. The results reveal that P-NDCX-1.0 had a better charge transfer rate than that of NDCX and S-NDCX-1.0.

The stability of P-NDCX-1.0/GCE in the reaction was evaluated by comparing the reduction overpotentials detected at the initial CV cycle and after 100 CV cycles, as shown in Figure 8F. The reduction overpotential detected after 100 CV cycles was −0.583 V vs. Ag/AgCl, which was only shifted by 14 mV compared with that detected at the initial CV cycle (−0.569 V vs. Ag/AgCl). Further analysis of the peak current and peak slope also demonstrated negligible changes between them (see the data presented in the picture). That is, the P-NDCX-1.0/GCE had good stability in the electrocatalytic reduction of p-NP.

## 3. Materials and Methods

### 3.1. Synthesis of Materials

*Synthesis of N-doped carbon xerogels.* The N-doped carbon xerogels were prepared according to previous reports [43,52,65]. Briefly, 9.09 g resorcinol, 0–4.0 g dicyandiamide, and 0.025 g Na_2_CO_3_ were added to 18 mL distilled water and heated to 90 °C with stirring, until the solids were completely dissolved. Then, the obtained solution was cooled to room temperature, and 12.4 mL formaldehyde was added dropwise. Thereafter, the mixture was aged at 85 °C for 72 h, yielding a solid gel, which was crushed and dried in an air oven at 100 °C for another 72 h. Finally, the material was heated in a N_2_ atmosphere with the following program: 150 °C for 75 min, 300 °C for 75 min, 600 °C for 150 min, and 800 °C for 100 min. The ramping rate between each temperature stage was 2 °C/min. For convenience, the N-doped carbon xerogel, prepared with addition of 2.0 g dicyandiamide, was named as NDCX.

*Synthesis of P or S doped NDCX.* A total of 0.2 g NDCX and 0.5–2.0 g diammonium hydrogen phosphate [(NH_3_)_2_HPO_4_] or 0.5–2.0 g thiourea (CH_4_N_2_S) were uniformly mixed and heated at 850 °C in a N_2_ atmosphere for 2 h, with a N_2_ flow rate of 50 mL/min. Based on the amount of (NH_3_)_2_HPO_4_ or CH_4_N_2_S added, the products were denoted accordingly as P-NDCX-m or S-NDCX-m, where “m” means the amount of [(NH_3_)_2_HPO_4_] or (CH_4_N_2_S) added.

### 3.2. Sample Characterization

Powder X-ray diffraction (XRD) measurements were determined on a Rigaku D/max TTR-III diffractometer in the 2θ range from 20° to 80°, using Cu Ka radiation (λ = 0.15405 nm). Fourier-transform Infrared (FTIR) spectra were obtained on a Vertex Perkin-Elmer 580B IR spectrophotometer (Bruker) with the KBr pellet technique. Transmission electron microscopy (TEM) images were collected on a FEI Tecnai TF30 apparatus. The powder was ultrasonically dispersed in ethanol before being deposited on the carbon-coated copper grid for observation. N_2_ physisorption isotherms were obtained on a BeiShiDe 3H-2000PS2 apparatus. The sample was degassed in vacuum at 300 °C for 5 h before measurement. The surface area was calculated with the Brunauer–Emmett–Teller (BET) method and the pore size distribution was calculated by the Nonlocal density functional theory (NLDFT) method. X-ray photoelectron spectra (XPS) were recorded on a VG ESCALAB MK II apparatus using Mg KR (1253.6 eV) as the X-ray excitation source. The binding energy was calibrated using the C1s signal of adventitious carbon at 284.6 eV.

### 3.3. Electrochemical Test

The electrocatalytic reduction of p-NP was carried out on a Shanghai Chenhua CHI636e electrochemical workstation with a standard three-electrode system, including a glassy carbon working electrode (GCE, L-type −3 mm), an Ag/AgCl reference electrode, and a platinum wire counter electrode. The reaction solution was composed of 0.2 mM p-NP in 0.2 M phosphate buffer solution (PBS, pH = 6.0). The working electrode was prepared as following: 4 mg of the acquired catalyst, 460 μL of isopropanol, and 40 μL of 0.3 wt% nafion solution were added to a 1 mL centrifuge tube and then ultrasonicated for 20 min to obtain a uniformly dispersed ink solution. Finally, 3 μL of the ink solution was dripped onto the GCE and dried in air at room temperature.

Cyclic voltammetry (CV) measurements were performed with a scanning rate of 20 mV/s between 0.4 V ~ and 0.9 V. The catalyst was activated before reaction to ensure that there were no impurities on the electrode surface, and it could be fully exposed to the electrolyte, guaranteeing the accuracy of the catalytic tests [66].

Electrochemical impedance spectroscopy (EIS) measurements of NDCX, P-NDCX-1.0, and S-NDCX-1.0 were taken using the controlled current method of the DH7000D electrochemical workstation. The frequency range was selected from 10^5^ to 0.01 Hz, the DC current was set to −0.0706 mA, and the amplitude was set to 0.007 mA, similar to the current during the test, corresponding to a current density of 1 mA/cm^2^.

For the stability test, the catalyst was continuously scanned for 100 cycles in the same potential range, with a scanning rate of 100 mV/s. The reduction peak potentials, before and after scanning, were compared to estimate the stability.

## 4. Conclusions

In summary, we reported the preparation of N,P-, and N,S- co-doped carbon xerogels with different dopings via the in situ vapor deposition method and used them as catalysts for the electrocatalytic reduction of p-NP. Among them, the sample P-NDCX-1.0 exhibited the best activity and excellent stability for the reduction reaction. Served as the precursor of the N atom, dicyandiamide possesses rich pyridine nitrogen offering abundant reactive sites for the reaction by donating electrons to the reactant. Because of the co-doping of these elements, the carbon xerogels have a suitable pore size and an applicable surface area, exposing a large number of active sites which can accelerate the rate the electrocatalytic reaction. These results demonstrated that the N and P co-doped carbon xerogels prepared by the current method could be promising catalysts for the electrocatalytic reduction of p-NP, with a low overpotential and favorable stability.

## Figures and Tables

**Table 1:** Textural properties of NDCX, S-NDCX-1.0, and P-NDCX-m obtained from the N_2_ physisorption isotherms.

	S_BET_ (m^2^/g)	V_mic_ (m^3^/g)	V_ful_ (m^3^/g)	D_mic_ (nm)	D_mes_ (nm)
0	655.9	0.30	0.59	0.54	7.73
0.5 g	612.8	0.28	0.51	0.53	7.71
1.0 g	585.6	0.30	0.72	0.50	9.84
NDCX	554.0	0.22	0.44	0.47	8.10
4.0 g	476.8	0.19	0.28	0.57	6.54
NDCX-0	594.2	0.29	0.51	0.49	8.27
P-NDCX-0.5	731.0	0.35	0.60	0.49	8.23
P-NDCX-1.0	989.3	0.47	0.75	0.57	8.31
P-NDCX-1.5	1277.7	0.61	0.97	0.53	7.83
P-NDCX-2.0	1522.1	0.72	1.14	0.53	6.84
S-NDCX-0.5	695.1	0.34	0.46	0.57	5.00
S-NDCX-1.0	644.6	0.32	0.53	0.53	8.27
S-NDCX-1.5	832.2	0.41	0.53	0.53	5.02
S-NDCX-2.0	788.5	0.39	0.51	0.54	5.00

S_BET_—BET surface area; V_mic_—micropore volume; V_ful_—total pore volume; D_mic_—micropore diameter; D_mes_—mesoporous diameter.

**Table 2:** Surface element percentage of NDCX, P-NDCX-1.0, and S-NDCX-1.0, detected from the XPS measurements.

	C (at. %)	O (at. %)	N (at. %)	P (at. %)	S (at. %)
NDCX	89.74	5.69	4.57	-	-
P-NDCX-1.0	89.12	6.37	4.50	0.02	-
S-NDCX-1.0	87.57	5.46	5.78	-	1.19

**Table 3:** The overpotential, peak current, and peak slope of blank GCE, NDCX, P-NDCX-1.0, and S-NDCX-1.0, obtained from the CV curves.

Catalyst	Peak Potential (V vs. Ag/AgCl)	Peak Current (μA)	Peak Slope (μA/V)
Blank GCE	−0.717	2.76	48.39
NDCX	−0.605	28.96	346.15
P-NDCX-1.0	−0.569	51.61	695.90
S-NDCX-1.0	−0.590	51.52	509.84

**Table 4:** Comparison of the activity of P-NDCX-1.0 for the electro-reduction of p-NP with other materials reported in the literature.

Catalyst	NP Conc.	Scan Rate (mV/s)	Peak Potential (V vs. Ag/AgCl)	Peak Current (μA)	Ref.
CuPd/C	10 mM	50	−0.94	127	[59]
FeO_x_/TiO_2_@mC	0.1 mM	50	−0.67	19.3	[60]
hollow globe WC	10 mM	50	−0.605	16.37	[61]
RGO/GCE	0.1 mM	50	−0.571	89.4	[4]
P-NDCX-1.0	0.2 mM	20	−0.569	51.61	This work

## Data Availability

Not applicable to this article.
